# Supplementary material for: Health impact of hepatic-venous-occlusive disease in a small town in Ethiopia—Case study from Tahtay koraro district in Tigray region, 2017
Source: PLoS One. 2019 Nov 5;14(11):e0224659. doi: 10.1371/journal.pone.0224659 (PMC6831030; doi:10.1371/journal.pone.0224659)
Supplement: Attachment — Submitted filename: Response to reviewers.docx [file pone.0224659.s002.docx]

**Revisions made on the manuscript to be submitted to Plos one**

**Health impact of hepatic-venous-occlusive disease in small town in Ethiopia – case study from Tahtay Koraro district in Tigray region, 2017**

**Version:** 1 **Date: 26 September,** **2019**

**Dear Editor-in-Chief:**

**Amendments made for the HVOD manuscript**

**Response to journal requirements’ comments**

1. We have addressed the Plos one’s style requirement
2. Ethical review board had waived the requirement for informed consent.
3. The minor occurrence of overlapping text with previously published journals was rewritten and edited.
4. The data set is uploaded as supporting information file
5. We have referred the figures in our text
6. We have referred the table in our text

**Response to reviewers’ comments**

1. The Title was modified to Health impact of hepatic-venous-occlusive disease in small town in Ethiopia – case study from Tahtay Koraro district in Tigray region, 2017 as commented.
2. The result of abstract has included trend over time of VOD fatality.
3. In line 29 we have added the problem after however
4. Page 3 line 53 was modified to - in different literatures it has been reported that the incidence of VOD….
5. The comment given on page 4, line 69; We have added additional literatures concerned on hepatic-venous-occlusive disease in Tahtay koraro district
6. On page 4 line 72 we have removed the sentence according to the given comment
7. On page 4, line 76 we have included the population size of Tahtay Koraro district in 2017
8. In the result section we have edited each tables and figures by describing fully
9. The typographical errors were edited
